# Depression Detection from Three-Channel Resting-State EEG Using a Hybrid Conv1D and Spectral–Statistical Fusion Model

**DOI:** 10.3390/s26051417

**Published:** 2026-02-24

**Authors:** Oana-Isabela Știrbu, Florin-Ciprian Argatu, Felix-Constantin Adochiei, Bogdan-Adrian Enache, George-Călin Serițan

**Affiliations:** 1 Doctoral School of Electrical Engineering, Faculty of Electrical Engineering, National University of Science and Technology Politehnica Bucharest (NUSTPB), 060042 Bucharest, Romania; 2 Department of Measurements, Electrical Apparatus and Static Converters, Faculty of Electrical Engineering, National University of Science and Technology Politehnica Bucharest (NUSTPB), 060042 Bucharest, Romania; florin.argatu@upb.ro (F.-C.A.); felix.adochiei@upb.ro (F.-C.A.); bogdan.enache2207@upb.ro (B.-A.E.)

**Keywords:** EEG, resting-state, major depressive disorder, depression detection, three-channel EEG, hybrid deep learning, feature fusion

## Abstract

**Highlights:**

**What are the main findings?**

- We propose a compact hybrid deep fusion model that combines a Conv1D representation of raw ≈15 s resting-state windows with per-channel spectral–statistical descriptors, trained with subject-independent (cross-subject) splits and class weighting to discriminate major depressive disorder from healthy controls using only three frontal EEG channels. The resulting model attains 93.43% window-level accuracy on the held-out MDD test subjects.
- The model is trained and selected on a single publicly available MDD dataset and then applied without additional adaptation to an independent three-channel UNICORN acquisition, providing a preliminary external feasibility check under a matched montage (Fp1–Fz–Fp2) and sampling rate (250 Hz).

**What are the implications of the main findings?**

- The findings suggest that low-burden, three-channel EEG, when paired with a lightweight hybrid architecture, may inform the design of scalable screening workflows and portable implementations; however, evidence beyond the training dataset remains preliminary and requires larger multi-site validation.
- The work provides a reproducible processing and modeling pipeline, from preprocessing and feature extraction to training and subject-level aggregation, designed to facilitate cross-dataset comparability and reduce dataset-specific preprocessing sensitivity, supporting future external validation across sites and transparent benchmarking.

**Abstract:**

Major depressive disorder requires scalable, low-burden screening tools. We examined whether three-channel resting-state EEG can support reliable discrimination between major depressive disorder and healthy controls using a lightweight model compatible with portable implementations. This work makes three main contributions: (i) a compact hybrid fusion model combining raw-window Conv1D embeddings with per-channel spectral–statistical descriptors for three-channel resting-state EEG, (ii) a leakage-resistant subject-independent (cross-subject) evaluation protocol with subject-level inference via majority voting, and (iii) a preliminary external feasibility test on an independent portable three-channel cohort without fine-tuning. The proposed model fuses a Conv1D encoding of raw ≈15 s eyes-closed windows (3840 samples; 15.36 s at 250 Hz) with per-channel spectral and statistical descriptors. Training uses subject-independent splits to avoid leakage, class weighting, and data augmentation (including MixUp); hyperparameters are selected via randomized search with refinement. The model is trained on a publicly available MDD dataset and subsequently applied, without fine-tuning, on an independent acquisition of 20 subjects recorded with a portable three-channel device; we report both window-level and subject-level (majority-vote) performance. On the held-out test subjects from the public dataset, the hybrid model attains 93.43% window-level accuracy. The independent evaluation is reported as a preliminary external feasibility analysis; given the small cohort, we report subject-level performance with 95% confidence intervals to reflect uncertainty and avoid over-interpreting cross-device generalization. The model occupies approximately 40.19 MB on disk, and the architecture is compatible with post-training int8 (TFLite) quantization for resource-constrained hardware. These results, obtained on limited samples, support the feasibility of three-channel EEG for major depressive disorder detection using a lightweight hybrid architecture and motivate prospective clinical validation, on-device inference and quantization studies, and broader evaluation across centers and devices.

## 1. Introduction

Major depressive disorder (MDD) is a leading cause of disability worldwide [1,2,3] and remains under-detected in primary and community settings, where time, expertise, and access to structured clinical interviews are limited [4]. Objective, scalable screening tools that can complement, rather than replace, clinician judgment are therefore of high interest. Electroencephalography (EEG) is particularly relevant in this context—it is non-invasive [5], relatively low-cost, and offers millisecond-level sensitivity to neural dynamics that may index affective dysregulation [6,7]. Although many EEG studies of depression have used dense montages and task-based paradigms, recent work suggests that short resting-state recordings from a minimal frontal montage [8] may capture discriminative information that is sufficient to support screening workflows [9,10].

In this study, we deliberately used a three-electrode frontal montage (Fp1, Fz, and Fp2) for two pragmatic reasons. First, the prefrontal cortex is central to emotion regulation and cognitive control, and resting frontal EEG markers (e.g., alpha asymmetry, theta dynamics) have repeatedly been reported to track affective dysregulation, supporting minimal-channel, frontally focused recordings for depression screening [11,12,13,14]. Consistent with this evidence, several three-channel studies suggest that frontal electrodes such as Fp1, Fz, and Fp2 can discriminate MDD from healthy controls (HC) with promising performance using either classical features or modern machine learning (ML), indicating that a compact frontal montage is viable for screening workflows [10,15]. Second, frontal placements minimize hair interference and reduce setup time, which is critical for portable and point-of-care use; recent work on minimal-channel and wearable EEG indicates that setup burden directly impacts feasibility and data quality [16,17]. In parallel, wireless research-grade amplifiers have matured: validation of the Unicorn Hybrid Black has shown frequency-domain measures comparable to laboratory systems and robustness suitable for out-of-lab acquisitions, which motivates our external, device-level evaluation without model retraining [18].

From a modeling standpoint, three broad families are commonly used. (i) Classical pipelines compute hand-engineered descriptors (e.g., relative band powers, theta/beta ratios, Hjorth parameters, entropy measures, and wavelets) and feed them to linear models, support vector machines (SVMs), k-nearest neighbors (KNNs), random forests, or boosting methods. These approaches are comparatively data-efficient and interpretable, and have shown competitive performance in MDD versus HC discrimination, including work on treatment-response prediction using wavelet-based features [19,20]. (ii) End-to-end deep-learning approaches learn directly from raw or minimally processed EEG, typically one-dimensional convolutional neural networks (1D-CNNs) or temporal CNN–recurrent neural network (RNN) hybrids, and in several studies have matched or exceeded feature-engineered or traditional ML baselines under subject-independent evaluation on public datasets [21]. (iii) Hybrid and transfer-learning approaches combine time–frequency representations (e.g., synchrosqueezed wavelets) with modern backbones (e.g., Residual Networks (ResNets)) or use domain-adaptation methods to improve robustness across heterogeneous datasets [22,23]. Collectively, the available evidence suggests that both engineered (feature-based) and learned (end-to-end) representations are viable, and that channel-efficient systems are feasible when acquisition, preprocessing, modeling, and evaluation are carefully tuned to real-world deployment constraints (e.g., limited channels, device variability, and ambient noise).

Despite progress, several open questions remain. First, generalizability can be overestimated when windowed segments from the same subject appear in both training and test folds. This leakage can be mitigated with group-aware (by-subject) cross-validation (CV), a subject-independent (cross-subject) protocol in which all windows from a participant remain within the same fold. In multi-site or multi-device settings, evaluations typically adopt an outer group-wise split (e.g., leave-one-site-out), with all model selection restricted to the training partition. This pitfall has been documented specifically for EEG deep-learning studies and, more broadly, for neuroimaging ML with small samples [24,25]. Second, studies diverge on which frequency bands are most informative: some report increased theta, while others decreased alpha, altered beta/gamma activity, or frontal asymmetries. These discrepancies are often linked to sample characteristics, preprocessing choices, and rest-versus-task paradigms; meta-analyses and reviews highlight the resulting inconsistency (e.g., mixed evidence for frontal alpha asymmetry) [26]. Third, aggressive artifact removal (e.g., independent component analysis (ICA) or artifact subspace reconstruction (ASR)) can improve signal quality yet also remove disorder-related variance or introduce analyst/device biases, an especially delicate trade-off with only three channels, where blind source separation is underdetermined [27]. Finally, device-level transfer remains uncommon: many models trained on public datasets are not evaluated on new hardware or independent acquisition sites, and when evaluated across databases, performance frequently declines, motivating prospective multi-site evaluations [28].

Recent EEG learning research has also explored more explicit mechanisms for fusing temporal and spatial information and for improving cross-domain generalization. For example, STRFLNet integrates multi-channel spatio-temporal representations via dynamic/static graph learning and hierarchical transformer-based fusion, and is evaluated under subject-independent protocols in EEG emotion-recognition benchmarks [29]. In parallel, cross-subject and cross-session transfer has been studied using multi-source domain adaptation frameworks such as FMLAN, which aligns labeled source domains to an unlabeled target domain through mutual learning and fine-grained alignment [30]. These approaches highlight active directions for leveraging richer spatial structure and domain adaptation in EEG. In contrast, the present study targets a low-channel (three-electrode) frontal montage and evaluates an independent dataset in a strictly held-out manner, motivating a lightweight fusion design that does not assume dense spatial graphs or access to target-domain data during training.

We investigate whether a three-channel frontal resting-state EEG setup can reliably discriminate MDD from HC using a lightweight hybrid model designed with portable deployment in mind. We fuse a one-dimensional convolutional (Conv1D) representation of short raw windows with a compact set of spectral–statistical descriptors, train under strict subject-independent splits, and develop the model exclusively on a publicly available MDD dataset. Although the original cohort comprises 64 participants (34 MDD, 30 HC), restricting the analysis to the eyes-closed (EC) condition and electrodes Fp1–Fz–Fp2 yields a final subset of 58 participants (30 MDD, 28 HC).

We then evaluate the trained model, without retraining, on an independent dataset of 20 subjects recorded with a portable three-channel device as a preliminary external feasibility assessment. With minimal preprocessing (per-recording z-scoring and no ICA/ASR), label-preserving augmentations, and subject-level aggregation via majority voting, the approach achieves robust performance on the held-out test subjects from the public dataset and is applied unchanged to the external recordings to assess robustness under a different acquisition pipeline; robust cross-device generalization requires larger, multi-site cohorts. Relative to prior three-channel studies that rely mainly on engineered features with classical learners, our experiments indicate that fusing raw-signal embeddings with spectral–statistical features improves performance over feature-only classical baselines under identical subject-independent evaluation and includes an external evaluation on a modern wireless EEG amplifier. Together, these elements define a reproducible, deployment-oriented pipeline and help clarify what three-channel EEG can currently offer for depression screening, while motivating prospective, multi-center studies on larger and more diverse cohorts. Accordingly, we summarize the main contributions as follows:

- A lightweight hybrid fusion architecture integrating raw-window Conv1D embeddings with compact per-channel spectral–statistical descriptors for three-channel resting-state EEG.
- A strict subject-independent (cross-subject) evaluation protocol that avoids window-level leakage, with hyperparameter selection confined to the development set and subject-level inference via majority voting as the deployment-relevant unit.
- A preliminary external feasibility evaluation on an independent three-channel dataset acquired with a portable wireless EEG device, applied without retraining/fine-tuning to assess robustness under a different acquisition pipeline.
- A deployment-oriented model characterization, including the final model’s disk footprint and compatibility with post-training int8 (TFLite) quantization for resource-constrained hardware.

## 2. Materials and Methods

### 2.1. MDD Dataset

This dataset comprised 64 participants (34 with MDD and 30 healthy controls, HC). MDD diagnoses were established according to DSM-IV criteria by qualified clinicians [31]. EEG was recorded with a 19-channel system (Fp1, F3, F7, Fz, Fp2, F4, F8, C3, C4, Cz, P3, Pz, P4, O1, O2, T3, T4, T5, and T6). Each recording session included a 5-min eyes-closed (EC) resting-state block, a 5-min eyes-open (EO) resting-state block, and a 10-min three-stimulus visual oddball task. Signals were sampled at 250 Hz with a 24-bit A/D resolution [19]. To align with our three-channel pipeline and ensure compatibility with the independent UNICORN dataset, we restricted analyses to electrodes Fp1, Fz, and Fp2 and to the EC resting-state condition. After applying these inclusion criteria and quality/availability checks, the final subset comprised 58 participants (30 MDD and 28 HC). This dataset was used solely for model development (training and validation under subject-independent splits); no models were tuned or evaluated on it using intra-subject or mixed subject–window folds.

### 2.2. UNICORN Dataset

The proprietary UNICORN dataset was recorded using a g.tec Unicorn Hybrid Black amplifier at 250 Hz with 24-bit A/D resolution [32,33]. The standard Unicorn EEG cap provides predefined sites (Fz, C3, Cz, C4, Pz, PO7, Oz, and PO8), but our study targeted only the frontal montage Fp1–Fz–Fp2 [34]. To ensure precise placement at these locations, we used a different EEG cap, the g.GAMMAcap2, which adheres to the extended 10–20 system and offers 86 intermediate positions [35]. Recordings were obtained in a resting-state, eyes-closed condition under controlled laboratory settings, and only the EEG from Fp1, Fz, and Fp2 was retained for analysis.

The sample comprised 20 participants (8 with major depressive disorder, MDD; 12 healthy controls, HC) and was used exclusively as an external test set. No model training, hyperparameter tuning, or decision-threshold calibration was performed on the UNICORN recordings. Given the limited cohort size (*n* = 20), UNICORN results are reported as a feasibility-oriented external test rather than definitive evidence of cross-device generalization. Psychological assessment included the Five-Factor Personality Inventory (FFPI; 100 items: Extraversion, Agreeableness, Conscientiousness, Emotional Stability, Autonomy) [36] and the Depression, Anxiety, and Stress Scales–21 (DASS-21; 21 items: Depression, Anxiety, Stress) [37]. Eligibility criteria required at least a 2-week washout from psychotropic medication, the absence of comorbid psychiatric disorders, and no organic brain lesions (e.g., epilepsy). Female participants with MDD confirmed non-pregnancy at enrollment. Exclusion criteria included lactation, current hormonal-contraceptive use, alcohol or psychotropic-substance abuse/dependence within the past year, and a history of abuse. To minimize acute stimulant/depressant effects, participants were asked to avoid coffee for 2 h and alcohol for 24 h before the session.

All procedures complied with the Declaration of Helsinki and the General Data Protection Regulation (GDPR) [38]. Participants were informed of their rights under GDPR (access, rectification, withdrawal of consent, restriction, and erasure where applicable), and written informed consent was obtained after providing detailed information about study aims, data handling, and participant rights. Data processing relied on explicit consent for scientific research and followed purpose limitation and data minimization: only variables essential to the objectives were retained (EEG at Fp1, Fz, Fp2; FFPI and DASS-21 scores; minimal demographics). Datasets were pseudonymized using random study identifiers, and the re-identification key was stored separately with access controls; participant identifiers are not permanent and can be reissued if procedural or regulatory needs arise.

#### EEG Data Acquisition Protocol

**Figure 1:**
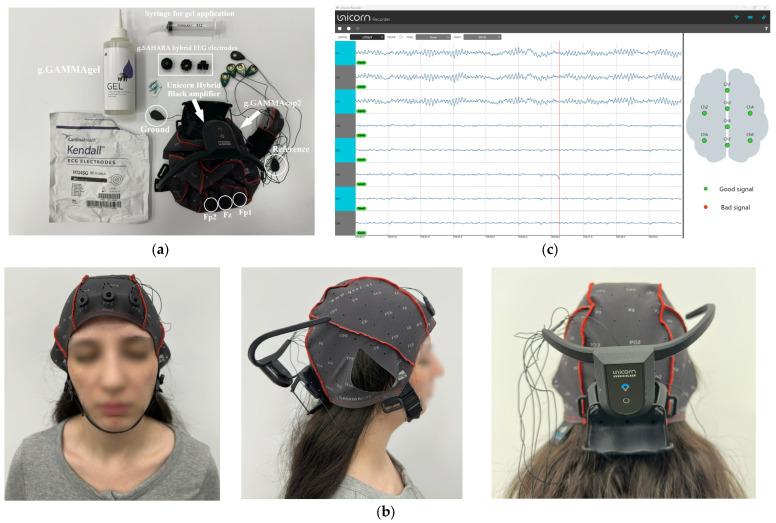
EEG data acquisition protocol: (**a**) hardware prior to mounting (g.GAMMAcap2, Unicorn Hybrid Black), highlighting Fp1–Fz–Fp2 targets and reference/ground; (**b**) cap fitted on participant (eyes closed), with impedances ≤ 20 kΩ; (**c**) recording interface during eyes-closed (EC) resting-state (250 Hz, 24-bit; built-in 50 Hz notch enabled during acquisition), with Fp1–Fz–Fp2 mapped to channels CH1–CH3. Green markers indicate good signal; red markers would indicate bad signal (none present in this recording). Additional channels are displayed by default but are not used for analysis; traces are shown in microvolts (typical EC alpha ≈ 20–100 µVpp).

The acquisition protocol is illustrated in Figure 1: (a) the hardware prior to mounting (g.GAMMAcap2 and the Unicorn Hybrid Black amplifier), with target sites Fp1–Fz–Fp2 and reference/ground leads highlighted; (b) the cap correctly fitted on a seated participant (upright posture, relaxed jaw, eyes closed), frontal electrodes seated, cable routing secured to minimize motion, and impedances within threshold (≤20 kΩ); (c) the Unicorn Recorder interface during eyes-closed resting-state recording, showing real-time traces at 250 Hz, 24-bit acquisition, with a 50 Hz notch filter enabled. Fp1, Fz, and Fp2 were mapped to channels CH1–CH3; additional channels remained visible in the software interface but were not used in monitoring or subsequent analysis. Recordings used g.SAHARA hybrid EEG electrodes (soft hybrid electrodes for dry or wet use, made of a biocompatible conductive polymer; 8 pins, 7 mm length; 20 mm diameter). In this study, wet recordings were performed using g.GAMMAgel (highly conductive, high-viscosity electrode gel for active electrodes; water-soluble, non-abrasive, non-greasy, non-irritant, non-corrosive; CE Class I).

Each session began with approximately 30 s stabilization, followed by a single ~90 s EC recording epoch (one continuous time window). Quality control required acceptable impedances and visual inspection for saturation or drift; recordings with more than 20% contamination were excluded. This configuration yields typical frontal morphology with alpha activity during EC and supports reproducibility via standardized placement, fixed sampling and filters, and explicit channel mapping.

### 2.3. Study Workflow

**Figure 2:**
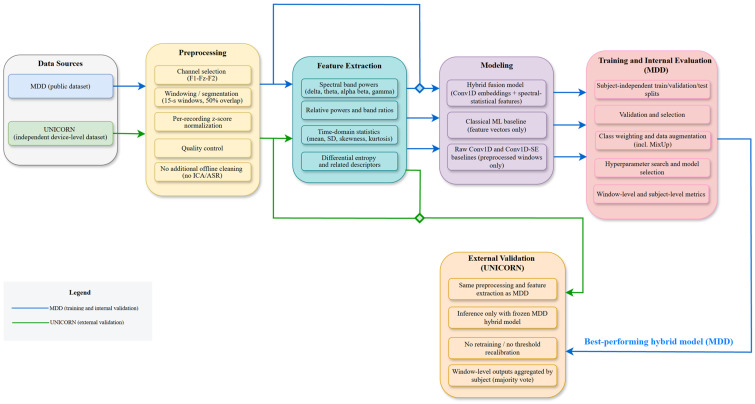
Study workflow. Major depressive disorder (MDD) data (.edf → .csv) and the UNICORN dataset for external validation undergo common preprocessing (Fp1–Fz–Fp2 selection, ≈15 s windows with 50% overlap, per-recording z-score, quality control; no ICA/ASR or extra offline filters). For MDD, preprocessed windows and derived spectral–statistical features feed three branches: Raw (Conv1D), Features → classical ML, and the Hybrid fusion model. The best-performing hybrid model trained with subject-independent splits is then applied without retraining to UNICORN using the same preprocessing and feature extraction, and performance is reported at the subject level via majority voting.

Figure 2 summarizes the end-to-end workflow used throughout the paper. After format harmonization (.edf → .csv) to a common three-channel representation (Fp1–Fz–Fp2), both datasets underwent an identical preprocessing pipeline that constrained analysis to the frontal montage, segmented the signal into fixed windows (length L, stride S, overlap p), and applied per-recording z-score standardization with basic quality control; no explicit artifact correction (ICA/ASR) or additional offline filtering was introduced.

For the MDD dataset, preprocessed windows either feed a raw Conv1D baseline directly or are passed to a feature-extraction stage that computes spectral and statistical descriptors. These feature vectors are used by classical machine-learning models and, together with the raw windows, by the proposed hybrid fusion model that combines raw-signal and spectral–statistical embeddings. Deep models are trained under strict subject-independent splits with class weights and light, label-preserving augmentations (including MixUp), and subject-level predictions are obtained by aggregating window outputs via majority voting. For external validation, we freeze the best-performing hybrid model trained on the MDD dataset and apply it, without additional fine-tuning, re-training, or threshold adjustment, to the UNICORN dataset using the same preprocessing and feature-extraction pipeline, followed by subject-level majority-vote aggregation.

### 2.4. Data Preprocessing

We applied a uniform preprocessing pipeline to both datasets (MDD and UNICORN), harmonizing recordings to the frontal montage Fp1–Fz–Fp2. To limit hand-crafted bias while preserving spectral structure relevant to depression screening, preprocessing was deliberately minimal: raw signals were z-scored per channel within each recording (mean removal and division by the within-recording standard deviation, SD). No explicit artifact removal (e.g., ICA/ASR), no manual epoch rejection, and no additional offline notch or band-pass filtering were applied. This choice reflects two considerations: (i) a three-channel frontal montage has insufficient spatial degrees of freedom for reliable blind source separation; and (ii) aggressive or parameter-sensitive cleaning can remove task-relevant variance and introduce dataset-specific bias [39,40]. Robustness was instead promoted via simple standardization and subject-level aggregation at evaluation, supporting cross-dataset comparability and deployment realism.

All recordings were processed using this template pipeline while preserving each dataset’s native sampling rate $f_{s}$. For the MDD dataset, EEG was originally stored as .edf files and exported to three-channel .csv files by selecting the frontal electrodes Fp1, Fz, and Fp2 before entering the main pipeline. UNICORN recordings were acquired directly as three-channel .csv files with Fp1, Fz, and Fp2 mapped to the first three columns. File-format conversion preserved the original time ordering of samples. Basic quality control removed windows with non-finite values (NaN/Inf), zero variance, or obvious clipping/saturation on import.

Recordings were processed at their native sampling rate $f_{s}=250\;Hz$ (no resampling). Each continuous EEG trace was segmented into fixed-length windows, parameterized in samples. We considered five nominal window durations (5, 10, 15, 20, and 24 s); at 250 Hz, these were implemented as 1344, 2560, 3840, 5040, and 6000 samples, corresponding to 5.37, 10.24, 15.36, 20.16, and 24.00 s, respectively. For readability, we refer to these operating points by their nominal durations (e.g., ≈15 s) while also reporting the exact sample count when needed (e.g., 3840 samples = 15.36 s). Stride S was set to achieve a target overlap p (tested values: 0, 10, 20, 30, 40, 50, 60, 70, 80, and 85%), where S=L×(1−p/100); window length in samples is L, and its equivalent duration is $L/f_{s}$. For the final operating point L=3840 samples and p=50%, the stride is S=1920 samples (i.e., 1920/250 s=7.68 s). The selected operating point is reported in Section 3.1. Recordings shorter than one full window were excluded. To ensure fair comparison, window length and overlap were selected on training folds only (group-aware, by-subject model selection on the development subjects). Hyperparameter selection was performed using only the development (training/validation) subjects; the held-out test subjects were not used during model selection and were evaluated only once for the final selected configuration. All stochastic components (e.g., initialization, shuffling, augmentation) were seeded for reproducibility (seed = 42).

Because the Conv1D architecture operates on discrete samples, the window length was tuned in sample counts rather than exact seconds. The candidate lengths were selected to approximate clinically interpretable durations (5, 10, 15, 20, and 24 s) while providing convenient input sizes for the convolution–pooling stack; the final model uses 3840-sample windows (15.36 s at 250 Hz; denoted as ≈15 s).

We used subject-independent (cross-subject) splits (grouped by participant), meaning all windows from a given subject are assigned to a single partition and subjects are disjoint across training/validation/test, to prevent leakage and inflated performance from overlapping, correlated windows [41]. After restricting the MDD dataset to the EC condition and electrodes Fp1–Fz–Fp2, 58 participants (30 MDD, 28 HC) were retained. A group-aware split (scikit-learn GroupShuffleSplit) was applied at the participant level, assigning ~80% of participants to a development set and the remaining ~20% to a held-out test set (used only for final evaluation). Within the development set, a second participant-level GroupShuffleSplit created separate training and validation subsets (~85%/~15% of the development set). This partitioning mirrors deployment to unseen individuals, avoids accuracy inflation from overlapping windows, and encourages learning disorder-related rather than person-specific patterns.

Window-level predictions were aggregated into a single label per subject via majority voting. For each subject, we counted the predicted class across all windows, and the class with the highest count determined the subject label (e.g., 7 MDD vs. 4 HC ⇒ MDD). In the event of an exact tie in vote counts, we resolved it deterministically by selecting the class returned by argmax over the vote-count vector. For descriptive purposes, we also report the mean per-subject MDD probability (0–1) obtained by averaging the window-level softmax outputs. Sliding-window segmentation with overlap provides a dense representation of each recording, but adjacent windows are not independent because they share samples (for 50% overlap, each adjacent pair shares half of the window). Accordingly, window counts reflect the granularity of the representation rather than the number of independent test samples. Accordingly, performance is summarized at the subject/recording level by aggregating window predictions via majority voting, while window-level metrics are reported as descriptive measures of within-recording consistency under the fixed segmentation protocol. Classification decisions, however, were based on majority voting over window-level predictions, without additional threshold optimization (e.g., Youden’s J) on validation subjects.

To mitigate class imbalance in the window-level training data, we applied class-dependent weights to the loss, assigning a higher weight to the minority class based on window counts in the training set. For the deep models, we used simple, label-preserving augmentations only on training windows. Specifically, we added zero-mean Gaussian noise with standard deviation 0.01 to the raw windows with probability 0.7, introduced occasional slow linear drifts by adding a channel-wise slope term up to approximately 3% of the signal amplitude with probability 0.3, and applied per-channel amplitude scaling with multiplicative factors drawn from $N(1.0,0.1^{2})$ with a probability of 0.5. MixUp augmentation was optionally applied within mini-batches via convex combinations of raw windows and their one-hot labels, using a Beta distribution with α=0.2. No augmentations were applied at validation or test time.

The augmentations used during training are intended to approximate modest sources of variability commonly encountered in short resting-state EEG segments recorded across sessions (e.g., residual broadband sensor noise, low-frequency baseline wander, and gain/impedance differences). Additive Gaussian noise provides a simple model of residual measurement noise after preprocessing. Slow linear drift introduces mild baseline variation over the window consistent with low-frequency wander. Channel-wise amplitude scaling models small multiplicative gain changes. MixUp forms convex combinations of two windows and their labels and is used here as a regularization strategy that encourages smoother decision boundaries in input space; it should be interpreted as interpolation-based regularization rather than as a physiologically faithful generative model of a single individual’s EEG. All augmentation magnitudes are kept small after per-recording standardization (z-scoring), and reported results are computed exclusively on unaugmented validation and test recordings.

### 2.5. Feature Extraction

We employed a unified, cross-dataset feature protocol applied identically to the MDD and UNICORN datasets so that differences in performance reflect data and model behavior rather than differences in feature computation. From each fixed, z-scored window, we computed the Welch power spectral density (PSD) per channel and integrated over canonical EEG bands: delta (0.5–4 Hz), theta (4–8 Hz), alpha (8–13 Hz), beta (13–30 Hz), and low/middle/high gamma (30–45/45–70/70–100 Hz) [42]. Welch estimates were obtained with a Hanning window of length $2f_{s}$, 50% overlap (segment length $2f_{s}$, overlap $f_{s}$), and detrending set to “constant”, and band powers were normalized by the total 0.5–100 Hz power of that window on each channel to obtain relative band-power profiles. From these, we derived canonical ratios summarizing spectral balance, computed by averaging relative band powers across channels and forming the following ratios: delta/low gamma, delta/middle gamma, theta/beta, theta/middle gamma, and alpha/low gamma.

To complement the spectral descriptors, we added per-channel time-domain statistics (mean, standard deviation, kurtosis, and skewness), as well as differential entropy. Differential entropy (DE) was approximated under a local Gaussian assumption as:

(1) $$h\approx \frac{1}{2}log(2\pi e\sigma^{2}),$$

where $\sigma^{2}$ is the variance of the z-scored samples within the window [43]. The resulting hand-crafted representation has two components. First, a per-channel matrix with eight values per channel, seven relative band powers (delta, theta, alpha, beta, and low/mid/high gamma) plus differential entropy, arranged as a 3 × 8 array that preserves channel identity (Fp1, Fz, and Fp2).

Second, we constructed a compact global window-level vector of 17 values that integrates two complementary sources of information: (i) twelve time-domain descriptors, given by the four distributional moments (mean, standard deviation, skewness, kurtosis) computed independently for each of the three frontal channels (4 × 3 = 12), and (ii) five spectral ratios derived from the relative band powers and chosen to capture commonly used spectral-balance contrasts (delta/low-gamma, delta/middle-gamma, theta/beta, theta/middle-gamma, and alpha/low-gamma). DE is not included in this global vector; it appears only in the per-channel 3 × 8 matrix as the eighth feature per channel, where it complements the relative band powers with a simple measure of residual dispersion after z-scoring.

The engineered feature set is designed to provide a compact, interpretable summary of resting-state spectral organization for a low-channel frontal montage [44,45,46,47]. Band-limited relative powers provide a coarse description of oscillatory activity across physiologically meaningful frequency ranges and are widely used in clinical EEG studies as a low-dimensional summary of resting-state dynamics [48,49,50]. Band ratios (e.g., θ/β, α/γ) complement absolute/relative band powers by encoding spectral balance in a scale-normalized form that is less sensitive to inter-subject amplitude differences and session-level gain effects [51]. DE provides an information-theoretic descriptor of within-window dispersion; under a standard local Gaussian approximation, DE is a monotonic transform of log-variance and therefore closely related to log band power [46,51]. We include powers/ratios and DE to capture complementary aspects of spectral magnitude, spectral balance, and distributional spread while keeping the feature space small and suitable for low-channel acquisition settings.

In the hybrid architecture, a shallow 1-D CNN encodes the 3 × 8 per-channel matrix, while a compact MLP transforms the 17-dimensional global vector; their embeddings are then concatenated with the raw-signal Conv1D embedding. The same 17-dimensional hand-crafted vector also serves as input for classical baselines (e.g., logistic regression (LR), SVM, random forest (RF), KNN, light gradient boosting machine (LightGBM), multilayer perceptron (MLP)), providing a compact, interpretable feature space shared across models and used consistently in the comparative analyses described in Section 2.7.

### 2.6. Model Architectures

This study focuses on a hybrid deep fusion model that integrates three complementary representations of each EEG window—raw time series, per-channel spectral–statistical features, and a compact global feature vector—and compares it against two baseline families: (i) classical feature-based models operating solely on the hand-crafted descriptors, and (ii) end-to-end Conv1D models trained directly on the preprocessed raw EEG.

#### 2.6.1. Hybrid Deep Fusion Model

**Figure 3:**
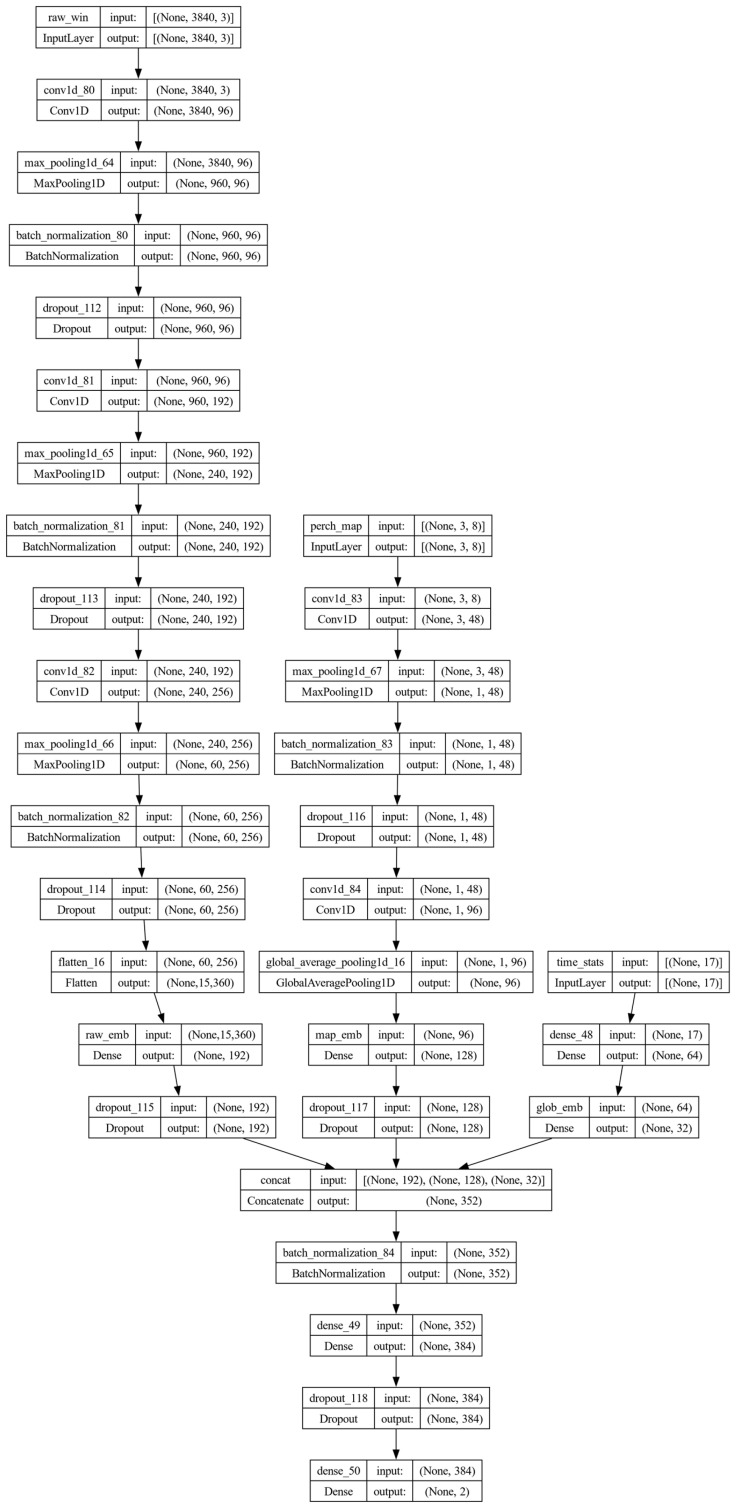
Architecture of the final hybrid Conv1D–feature fusion model (selected configuration: Trial 4 after Stage-2 refinement; Section 3.1). The network has three inputs: (i) raw 3-channel EEG windows (*L* = 3840 samples at 250 Hz; 15.36 s, denoted as ≈15 s; size *L* × 3), (ii) the 3 × 8 per-channel spectral–statistical matrix (seven relative band powers plus differential entropy (DE) per channel), and (iii) a 17-dimensional global feature vector (time-domain statistics and spectral ratios). The raw branch comprises three Conv1D blocks with 96, 192, and 256 filters, each followed by MaxPooling1D (pool size 4), batch normalization, and dropout, reducing the temporal length 3840 → 960 → 240 → 60 (=3840/4^3^); the output is flattened and projected to a 192-unit embedding (raw_emb). The per-channel feature branch applies Conv1D (48 filters) + MaxPooling1D, then Conv1D (96 filters) with global average pooling and a 128-unit dense layer (map_emb). The global feature branch is a two-layer MLP with 64 and 32 units (glob_emb). The three embeddings are concatenated and passed through a 384-unit dense fusion layer with dropout and a final 2-unit softmax output (major depressive disorder (MDD) versus healthy controls (HCs)).

The proposed hybrid deep fusion model combines complementary representations of each fixed-length window. The architecture comprises three parallel branches: a Conv1D branch operating directly on the preprocessed raw EEG (L×3), a shallow Conv1D branch applied to the per-channel spectral–statistical matrix (3 × 8), and a compact MLP applied to the 17-dimensional global feature vector. In addition to the raw EEG branch, the model incorporates compact frequency-domain summaries: a per-channel spectral–statistical map (relative band powers and differential entropy) processed by a lightweight Conv1D subnetwork, and spectral ratios included in the global feature vector. This design combines learned representations from raw signals with physiologically motivated spectral information while keeping input dimensionality and overall model complexity low. In this formulation, the Conv1D branch learns time-domain morphology and inter-channel interactions from raw windows, while the engineered-feature branches provide low-dimensional, scale-normalized spectral summaries. The resulting three embeddings are concatenated and passed to a fusion head (fully connected layers with dropout and a final softmax layer) that performs the binary MDD versus HC classification. We selected concatenation as a simple, compact, and widely used fusion operator that preserves information from each branch while adding minimal parameters, consistent with our emphasis on lightweight deployment and the limited number of subjects available for training. More expressive fusion schemes (e.g., gated/weighted fusion or attention/bilinear interactions) could be explored in future work as larger multi-site datasets become available. In higher-density EEG settings, recent work has used graph-based spatio-temporal encoders and transformer fusion to integrate multi-channel representations (e.g., STRFLNet) [29], and multi-source domain adaptation to improve cross-subject/cross-session transfer (e.g., FMLAN) [30]. Adapting such mechanisms to low-channel depression screening, where spatial structure is limited and independent target-domain training data may not be available, remains an important direction for larger cohorts. The complete layer-by-layer architecture of the final selected configuration is shown in Figure 3.

The raw EEG branch receives a z-scored window of shape L×3, where L denotes the number of samples per window. In the final configuration, we used *L* = 3840 samples at 250 Hz (15.36 s), denoted throughout as ≈15 s. The value 3840 was chosen so that three successive max-pooling layers with a pool size of 4 reduce the temporal dimension from 3840 to exactly 60 samples ($3840/4^{3}=60$), avoiding implicit padding or truncation. This branch acts as a temporal feature extractor and consists of three one-dimensional convolutional blocks with kernel sizes 11, 7, and 5, respectively.

Each block applies a Conv1D layer with “same” padding, ReLU activation, and $l_{2}$ regularization, followed by max-pooling (pool size 4), batch normalization, and dropout, progressively reducing temporal resolution while increasing the number of feature maps. The final feature map is flattened and projected into a dense layer with ReLU activation, yielding a compact raw-signal embedding (raw_emb), after which a fixed dropout of 0.50 is applied. The dimensionality of raw_emb is controlled by a hyperparameter (emb_raw), while the depth and width of the raw branch are governed by a three-element filter tuple (raw_filters) and a corresponding three-element dropout tuple (raw_drop), which specify the number of filters and dropout probabilities for each of the three Conv1D blocks. These hyperparameters (emb_raw, raw_filters, raw_drop), together with the initial learning rate (lr_init), weight decay (wd), and the size of the fusion-head hidden layer (head_units), are tuned by randomized search with refinement, as summarized in Section 3.1.

The per-channel feature branch operates on the 3 × 8 matrix constructed in Section 2.5 (seven relative band powers and one DE value per channel). This branch is implemented as a shallow two-layer Conv1D network applied along the feature dimension, with Conv1D layers (kernel size 3, “same” padding, ReLU activation, and $l_{2}$ regularization) interleaved with max-pooling (pool size 2), batch normalization, and dropout. A global average pooling layer aggregates across the remaining spatial dimension, and a dense layer with 128 units and ReLU activation produces the spectral-feature embedding (map_emb), followed by fixed dropout. The number of filters in the two Conv1D layers and the dropout probabilities in this branch are controlled by the map_filters and map_drop hyperparameters and are tuned by the same randomized search with refinement used for the raw branch, whereas the 128-dimensional size of map_emb is fixed a priori and not optimized during hyperparameter search.

The global feature branch operates on the 17-dimensional vector comprising 12 time-domain statistics (four distributional moments per channel) and 5 spectral ratios. A small MLP with two fully connected layers transforms this descriptor into the global embedding (glob_emb). Concretely, the input vector (G=17) is fed to a Dense layer with 64 units and ReLU activation, followed by a second Dense layer with 32 units and ReLU activation, both with $l_{2}$ regularization on the weights. The output of the second layer defines the 32-dimensional glob_emb. These layer sizes (64 and 32 units) are fixed by design rather than optimized during hyperparameter search. This branch injects interpretable, low-dimensional information into the network, stabilizes training, and provides an inductive bias towards capturing global spectral–temporal balance at the window level.

In the fusion head, the three embeddings (raw_emb, map_emb, glob_emb) are concatenated and passed through batch normalization and a fully connected hidden layer with ReLU activation, followed by dropout. The size of this hidden layer (head_units) is treated as a hyperparameter. The output layer is a 2-unit softmax corresponding to the MDD and HC classes. All convolutional and dense layers use $l_{2}$ weight regularization. Overall, the depth of each branch and the dimensions of map_emb (128 units) and glob_emb (32 units) are fixed as described above, whereas the raw-branch embedding size, convolutional filter counts, head hidden units, dropout rates, initial learning rate, and weight decay are tuned within a predefined search space, summarized in Section 3.1.

#### 2.6.2. Classical Feature-Based Models

Classical machine-learning baselines operated exclusively on the hand-crafted features described in Section 2.5. For each window, the 17-dimensional global descriptor (12 time-domain statistics and 5 spectral ratios) was standardized using a scaler fitted only on the training subjects and then applied unchanged to validation and test subjects, and the resulting features served as input to LR, SVM, KNN, RF, gradient boosting (GB), MLP, LightGBM, and a soft-voting ensemble combining LR, SVM, RF, GB, and MLP. All models were implemented in scikit-learn and LightGBM (Python), with seed = 42.

Key hyperparameters were tuned on the training subjects only, using simple grid or random searches under the same subject-independent splits as the deep models. For LR and SVM, we varied the regularization strength C∈{0.01,0.1,1,10,100} (L2 penalty, liblinear/‘lbfgs’ solver for LR; RBF kernel for SVM with $\gamma \in \{10^{-3},10^{-2},10^{-1},1\}$), KNN used k∈{3,5,7,9} with Euclidean distance, and RF/GB/LightGBM explored numbers of trees in {100,200,500} and maximum depth in {3,5,7}. The MLP baseline used one hidden layer with {32,64,128} units, ReLU activation, $l_{2}$ regularization, and early stopping. For models that support it (e.g., LR, SVM, RF, GB), we enabled automatic inverse-frequency class weighting to compensate for class imbalance. Classifiers were trained and evaluated at the window level, then aggregated to the subject level by majority voting as described in Section 2.4. The exact hyperparameter grids and best validation configurations for each model are reported in Table 1.

#### 2.6.3. Conv1D-Based Deep Baselines

To contextualize the hybrid model, we also trained end-to-end Conv1D baselines that operate directly on raw EEG windows. These models take the z-scored L×3 waveform as input (windows segmented as described in Section 2.4) and follow the same overall pattern as the raw branch of the hybrid architecture: a small stack of one-dimensional convolution and pooling layers, followed by a fully connected classifier head implemented with standard Keras layers.

The first baseline (“Conv1D-raw”) uses three Conv1D blocks with kernel sizes of 11, 7, and 5 and 64, 128, and 256 filters, respectively. All convolutions use “same” padding, ReLU activation, and $l_{2}$ weight regularization. Each block is followed by max-pooling (pool size 4), batch normalization, and dropout (0.25, 0.35, and 0.45 across the three blocks). The resulting feature map is flattened and passed to a dense layer with 256 ReLU units (with $l_{2}$ regularization), followed by dropout (0.50) and a final 2-unit softmax output layer. Optimization used the Adam optimizer with an initial learning rate of $8\times 10^{-4}$ and sparse categorical cross-entropy loss. Training was performed under the same subject-independent splitting strategy as the hybrid model (development versus held-out test subjects with a subject-independent validation split within the development set), and class weights were derived from the training window distribution to mitigate imbalance, with performance reported at both window and subject level via majority voting.

The second baseline (“Conv1D-SE”) augments this backbone with squeeze-and-excitation (SE) blocks after each convolutional layer. Each SE block performs global average pooling across time, followed by a two-layer gating MLP with a reduction ratio r=8 and a sigmoid output, which rescales the feature maps channel-wise through multiplicative weights. Apart from these SE modules, Conv1D-SE uses the same three Conv1D blocks (64, 128, and 256 filters; kernel sizes of 11, 7, and 5; max-pooling with a pool size of 4; batch normalization; dropout of 0.25/0.35/0.45) and the same 256-unit dense layer before the softmax output. This model was trained with Adam (initial learning rate $10^{-3}$) and sparse categorical cross-entropy, using the same subject-independent splits as Conv1D-raw. For Conv1D-SE, we additionally employed an on-the-fly augmentation generator that, for each training window, applies three operations with probability 0.5 each: (i) additive zero-mean Gaussian noise with standard deviation 0.02 in z-scored units, (ii) circular temporal shifts by a random offset drawn uniformly from [−0.1L,0.1L] samples (up to 10% of the window length), and (iii) per-channel amplitude scaling by factors sampled from $N(1.0,0.05^{2})$. Aligning the convolution–pooling structure and evaluation protocol of these Conv1D baselines with the hybrid model ensures that performance differences primarily reflect the contribution of feature fusion (raw signal plus spectral–statistical branches) and SE-based channel attention rather than differences in preprocessing or data partitioning. The best-performing configurations for the Conv1D-raw and Conv1D-SE baselines, including all selected hyperparameters, are summarized in Table 1.

### 2.7. Training and Evaluation Protocol

#### 2.7.1. Experimental Setup and Training Environment

Experiments were implemented in Python 3.10.11 using TensorFlow 2.10.0 (Keras API, DirectML backend), scikit-learn 1.7.2, NumPy 1.26.4, SciPy 1.15.3, pandas 2.3.3, and Matplotlib 3.10.7. Training was performed on a Lenovo ThinkPad P16 Gen 1 mobile workstation running Windows 11 Pro (version 25H2, build 26200.7171), equipped with a 12th Gen Intel Core i9-12900HX CPU (16 cores, 24 threads), 32 GB DDR5 RAM, a 1 TB PCIe 4.0 NVMe SSD, and an NVIDIA RTX A4500 Laptop GPU (16 GB GDDR6). TensorFlow (DirectML backend) exposed the RTX GPU as two DirectML devices, allowing the Conv1D baselines and the hybrid Conv1D–feature model to be trained on the GPU and to exploit data-parallel convolution and dense-layer operations, which reduced wall-clock time and enabled larger batch sizes and broader hyperparameter sweeps. DirectML was used to access the GPU natively on Windows without a CUDA toolchain, improving portability across hardware vendors on this platform. All experiments used a fixed random seed of 42 for Python, NumPy, and TensorFlow. Classical learners (SVM, RF, KNN, MLP, GB, and LightGBM) were trained on the CPU, as their compute profiles did not benefit meaningfully from GPU acceleration in this setting.

#### 2.7.2. Classification Pipelines and Training Protocol on MDD

We benchmarked a focused family of classifiers on the MDD versus HC task, spanning (i) classical learners over hand-crafted EEG features and (ii) temporal Conv1D models that operate directly on raw three-channel windows, including a hybrid fusion of both.

Train/validation/test partitions were created using group-aware (by-subject) splitting (i.e., subject-independent/cross-subject), so no subject appears in more than one set (see Section 2.4). Under the subject-independent split, classical pipelines with the 17-dimensional feature vector already reached high window-level accuracies (≈89–93%; Table 1), indicating that the compact descriptors are informative under strict subject-independent evaluation. The hybrid Conv1D–feature model is the best-performing single model on the MDD test windows, with a window-level accuracy of 93.43% (Table 1, Section 3.1), and yields consistent subject-level predictions under majority voting; however, uncertainty remains substantial because the held-out test cohort is relatively small.

With the small held-out test set, subject-level accuracy changes in 8.33% increments, and several model families attain the same subject-level score. We therefore also report window-level metrics as a complementary, higher-resolution summary of within-subject consistency under a fixed segmentation protocol, while interpreting subject-level voting as the primary deployment-relevant outcome.

For classical baselines, we trained LR, SVM with radial basis function kernel (SVM-RBF), KNN, RF, GB, LightGBM, a shallow MLP on features, and two ensembles (a soft-voting ensemble and a stacking model; Table 1). The 17-dimensional feature vector was standardized using a StandardScaler fitted on the training subjects and then applied to validation and test subjects. No augmentations were applied in feature space to avoid artificially altering the feature distribution. Instead, we mitigated class imbalance by applying inverse-frequency class weighting in the loss function where supported, together with $l_{2}$ regularization, early stopping, and probability calibration when applicable. All classical models were trained and evaluated at the window level and then aggregated to the subject level using majority voting over windows from the same subject.

For the hybrid model, inputs were z-scored windows of *L* = 3840 samples (15.36 s at 250 Hz) with 50% overlap (Section 3.1). For the Conv1D-raw and Conv1D-SE baselines, window length was treated as a tunable preprocessing parameter over the candidate set in Section 2.4, and the best-performing configurations (including their selected window lengths) are reported in Table 1. For the hybrid model, training windows were augmented using simple label-preserving operations applied in the time domain: additive zero-mean Gaussian noise (*σ* = 0.01 in z-scored units), occasional slow linear drifts implemented as channel-wise slopes up to ≈3% of the signal amplitude, per-channel amplitude scaling with factors drawn from 𝒩(1.0, 0.1^2^), and optional MixUp within mini-batches (Beta(α = 0.2, α = 0.2)). Validation and test windows were not augmented. Additional details on the interpretation and constraints of the augmentation operators are provided in Section 2.4. Hyperparameters of the hybrid architecture (Conv1D filter counts and dropouts, raw-branch embedding size, learning rate, and weight decay, as well as fusion-head width) were tuned on training and validation subjects only using a custom randomized search (12 trials) followed by refinement of the top-3 configurations, with val_accuracy as objective and early stopping plus ReduceLROnPlateau during the search. The best configuration was then retrained once on the combined training and validation subjects and finally evaluated on the held-out test subjects.

For comparison, we trained two end-to-end Conv1D baselines under the same preprocessing pipeline and subject-independent split: a plain Conv1D model and a Conv1D-SE model. The Conv1D-SE model used on-the-fly augmentations consisting of Gaussian noise, circular temporal shifts of up to 10% of the window length, and mild channel-wise amplitude scaling, whereas the plain Conv1D model was trained without data augmentation and with a manually specified configuration (no systematic hyperparameter search). We deliberately restricted the model family to architectures that (i) match our two input regimes (tabular features versus raw windows), (ii) have compact, reproducible hyperparameter spaces that can be explored under limited data, and (iii) remain stable under subject-independent splits. Heavier sequence models (e.g., deep transformers or stacked RNNs) were not considered, given the three-channel setting, limited sample size, and associated risk of overfitting and miscalibrated predictions.

At both window and subject levels, we report overall accuracy, class-wise precision, recall, F1-score, and balanced accuracy, together with the Matthews correlation coefficient (MCC). Accuracy is accompanied by 95% confidence intervals computed using the Wilson score interval for binomial proportions, which provides more reliable coverage than the normal (Wald) approximation for moderate sample sizes.

## 3. Results

### 3.1. Final Model Selection and Performance on the MDD Dataset

Model selection on the MDD development subjects identified a window length of 15.36 s (*L* = 3840 samples at 250 Hz) and 50% overlap (*p* = 50%; stride *S* ≈ 7.7 s) as the best-performing configuration. The final MDD model is the hybrid architecture shown in Figure 3, combining a Conv1D branch on raw three-channel windows with branches operating on the engineered spectral–statistical features. We used a two-stage model selection procedure: a short-run randomized search to identify promising configurations (Table 2), followed by longer refinement retraining of the top candidates (Table 3). Specifically, we ran 12 randomized-search trials over architectural and optimization settings (raw-branch embedding size, fusion-head width, initial learning rate, dropout rates, convolutional filter counts, and weight decay) and then retrained the three best-performing short-run configurations for longer in a refinement stage. The full set of Stage-1 validation accuracies and corresponding hyperparameters is summarized in Table 2, and the three Stage-2 refinement runs are reported in Table 3.

**Figure 4:**
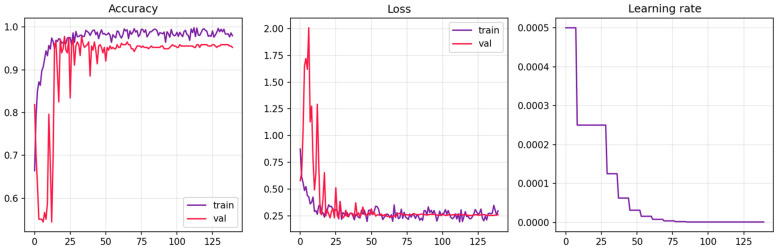
Training and validation accuracy, loss curves, and learning-rate schedule for the final hybrid model on the major depressive disorder (MDD) dataset.

**Figure 5:**
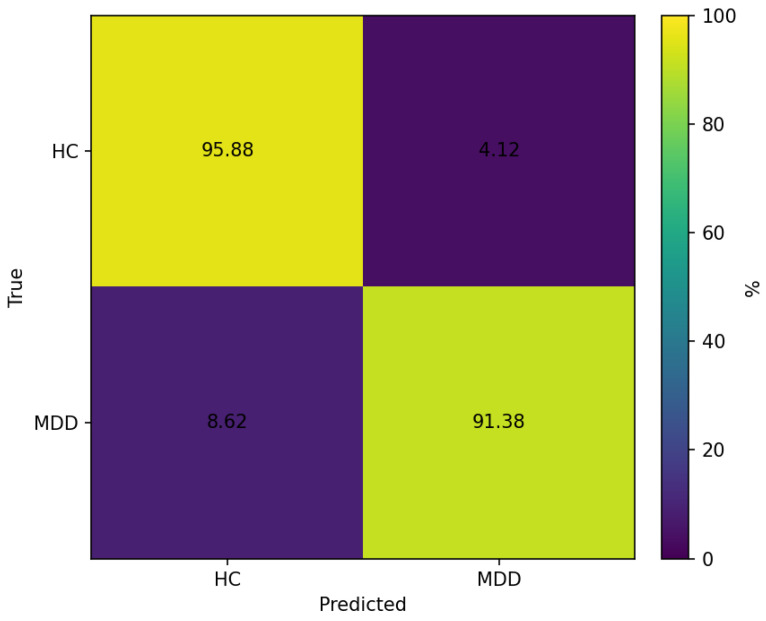
Window-level confusion matrix (percentages) for the final hybrid model on the major depressive disorder (MDD) test set (overall window-level accuracy 93.43%).

Among the 12 randomized-search trials, Trial 10 reached the highest validation accuracy in the initial short-run search (99.36%). However, when this configuration was retrained longer in the refinement stage, its validation accuracy dropped to 74.20%, indicating that the short-run peak was not stable under extended optimization. In contrast, Trial 4 improved from 86.62% to 98.73% during refinement and showed stable training/validation curves (Figure 4). We therefore selected Trial 4 based on refinement-stage validation performance and stability. To avoid selection bias, the held-out test subjects were not used for hyperparameter selection and were evaluated only once, after the final configuration was fixed. On the held-out test cohort, we aggregated window predictions into one decision per subject via majority voting, yielding perfect subject-level accuracy. Because subject-level evaluation is based on a limited number of independent test participants, the corresponding 95% Wilson confidence interval is wide, and the results should be interpreted descriptively. With no subject-level errors observed, the corresponding subject-level balanced accuracy is 100.0%, and the MCC is 1.00. For completeness, the same configuration attains 93.43% window-level accuracy under the fixed sliding-window representation (Figure 5; Table 4). Trial 6 reached 97.45% validation accuracy after refinement but did not offer a clear advantage over Trial 4 in refinement-stage validation, model size, or runtime. We therefore selected the Trial 4 configuration as the final hybrid model for all subsequent analyses, including external evaluation on the UNICORN dataset.

Figure 4 illustrates the training behavior of the selected hybrid model on the MDD development set. Training and validation accuracy increase rapidly over the first ~20–30 epochs and then plateau with only modest fluctuations, without a widening gap between the two curves. The corresponding loss trajectories show a gradual decrease and remain stable over later epochs. The learning-rate curve follows a stepwise decay schedule, with reductions triggered after short plateaus in validation accuracy and enabling finer convergence thereafter. Taken together, the coupled train/validation curves and controlled learning-rate decay suggest that the final configuration trains stably and does not exhibit marked overfitting on the development data.

Figure 5 summarizes the window-level test performance of the hybrid model under the strict subject-independent split. Out of 426 windows (HC = 194; MDD = 232), the model attains an overall accuracy of 93.43%. Although the recording protocol includes a 5 min EC block, the window count reflects the effective EC duration retained per recording after start/end trimming and quality-control exclusion, followed by ≈15 s windowing with 50% overlap. Because these windows are generated with 50% overlap, adjacent windows share samples and are correlated; window-level performance, therefore, provides a high-resolution view of within-subject consistency rather than performance over 426 independent test cases. Class-wise, 95.88% of HC windows are correctly identified (186/194), whereas 91.38% of MDD windows are correctly detected (212/232). Errors are slightly asymmetric, with more MDD windows misclassified as HC (8.62%) than HC windows misclassified as MDD (4.12%), indicating a tendency toward higher specificity than sensitivity. In addition to window-level metrics, we summarize performance at the subject level by majority voting across windows (Table 1), which is the deployment-relevant decision rule used in this study. Table 4 reports the full set of window-level metrics: accuracy 93.43% (Wilson 95% CI 90.66–95.41%), class-wise precision of 0.90 (HC) and 0.96 (MDD), recall of 0.96 (HC) and 0.91 (MDD), and F1-scores between 0.93 and 0.94. The balanced accuracy is 93.63%, and the MCC (Equation (2)) is 0.87, indicating substantial agreement between window-level predictions and labels under the fixed sliding-window representation. These window-level confidence intervals quantify uncertainty over the windowed representation; subject-level uncertainty is governed by the number of independent test participants.

The MCC was used as a summary measure that incorporates all four entries of the confusion matrix. For binary classification, it is defined as

(2) $$MCC=\frac{TP\cdot TN-FP\cdot FN}{\sqrt{\left(TP+FP)(TP+FN)(TN+FP)(TN+FN\right)}}$$

where TP, TN, FP, and FN denote true positives, true negatives, false positives, and false negatives, respectively [52]. MCC ranges from −1 (complete disagreement between predictions and labels), through 0 (chance-level performance), to +1 (perfect agreement), providing a balanced indicator of performance even under class imbalance.

Across models, window-level test accuracies on the MDD dataset fall within a relatively narrow range (≈89–93%; Table 1). Note that the best-performing Conv1D baselines in Table 1 used different window lengths from the hybrid model (Conv1D-raw: 10.24 s; Conv1D-SE: 24.0 s), reflecting that window duration was treated as a tunable preprocessing parameter over the candidate set in Section 2.4. This suggests that, under the present preprocessing and feature protocol, the MDD versus HC labels are reasonably separable and that several distinct model families can exploit this structure. The hybrid deep fusion model attains the highest window-level accuracy (93.43%), but the margin over well-tuned classical and Conv1D baselines is modest. Given the limited number of independent held-out test participants and the within-subject correlation induced by overlapping windows, differences between models are reported descriptively, and we therefore do not claim statistical superiority of the hybrid model over strong baselines. Subject-level majority voting provides a deployment-relevant per-participant summary that may differ even when window-level accuracies are similar.

### 3.2. Demographic Comparability and Psychometric Validity for UNICORN Dataset

**Figure 6:**
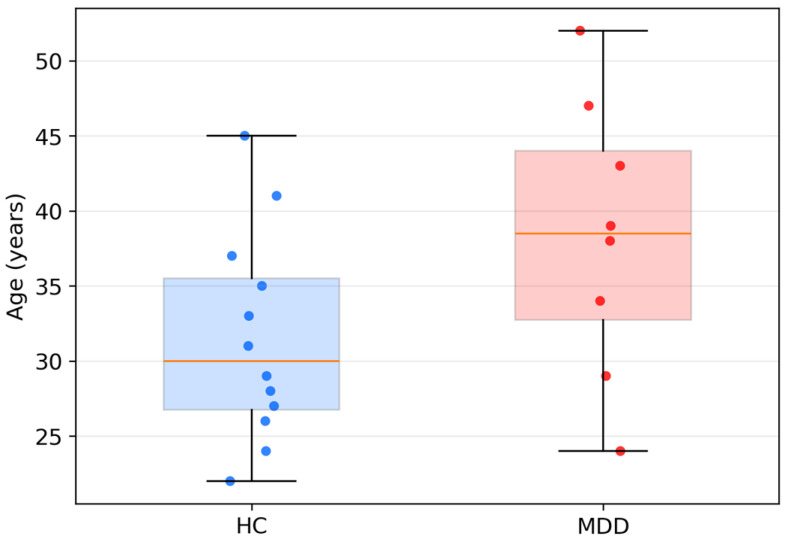
Age by group. Boxplots and individual data points for age in healthy controls (HC, blue) and participants with major depressive disorder (MDD, red) in the UNICORN dataset. The orange line indicates the median age within each group. Group differences are not statistically significant (Mann–Whitney p ≈ 0.097; Welch’s *t*-test p ≈ 0.103).

**Figure 7:**
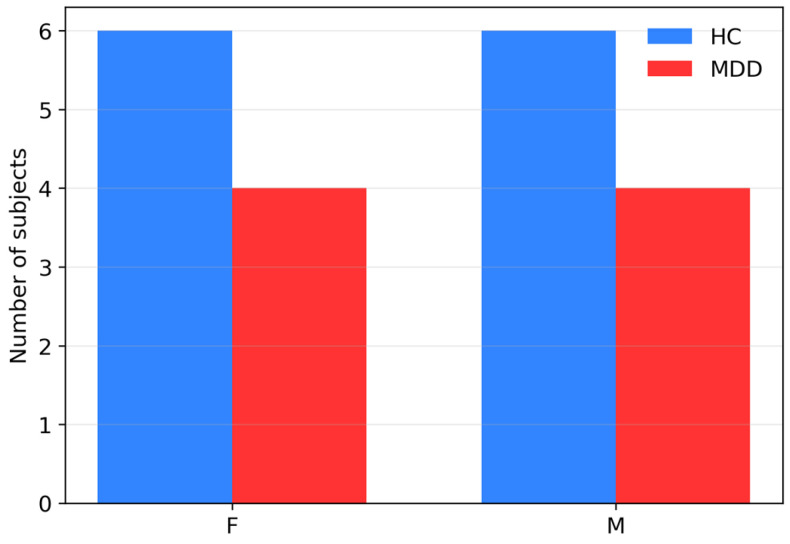
Gender by group. Counts of females (F) and males (M) in the healthy controls (HC, blue) and major depressive disorder (MDD, red) groups in the UNICORN dataset. There is no evidence of gender imbalance between groups (χ^2^ p = 1.00).

**Figure 8:**
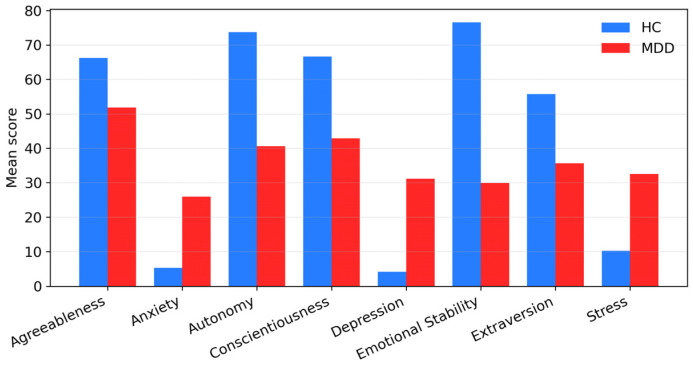
Questionnaire means by group. Mean scores for the FFPI traits (Extraversion, Agreeableness, Conscientiousness, Emotional Stability, Autonomy) and the DASS-21 subscales (Depression, Anxiety, Stress) for healthy controls (HC, blue) and major depressive disorder (MDD, red) participants. Higher Emotional Stability reflects lower neuroticism [54].

We summarized subject-level demographics and questionnaire scores for the UNICORN dataset (12 HC, 8 MDD). The groups were broadly comparable in age (HC: *n* = 12, mean 31.5 years; MDD: *n* = 8, mean 38.3 years), with no statistically significant difference on either the Mann–Whitney test (p ≈ 0.097) or Welch’s *t*-test (p ≈ 0.103), and showed no evidence of gender imbalance (χ^2^ p = 1.00; Figure 6 and Figure 7). Psychometric scores differentiated the groups in clinically expected directions (Figure 8). On the DASS-21, MDD participants had higher Depression, Anxiety, and Stress scores than HCs (all Mann–Whitney p ≈ 2.4 × 10^−4^). On the FFPI, MDD participants showed lower [53] Emotional Stability, Extraversion, Conscientiousness, Autonomy, and Agreeableness (all Mann–Whitney p ≈ 1.6 × 10^−5^). Welch’s *t*-tests yielded directionally consistent and highly significant differences (e.g., Depression p ≈ 1.0 × 10^−8^). Overall, the sample is demographically balanced and psychometrically well differentiated, supporting its use as a preliminary external test set for the hybrid model, despite its small size.

### 3.3. Validation with UNICORN Dataset

**Figure 9:**
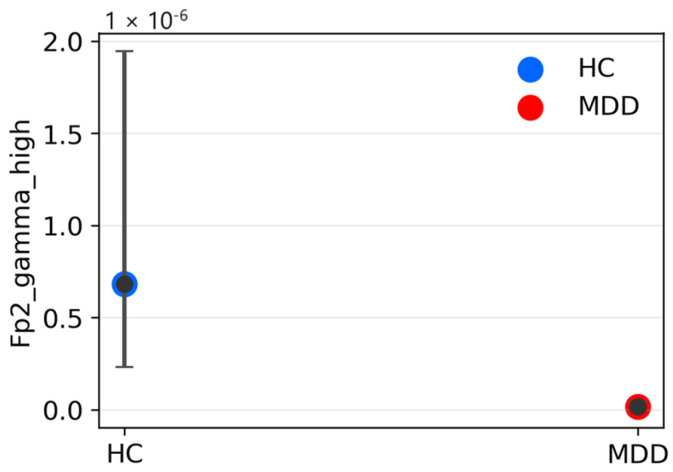
Subject-level comparison for Fp2 relative high-gamma power in the UNICORN dataset. Mean ± 95% bootstrap confidence interval of Fp2 relative high-gamma power for healthy control (HC, blue) and major depressive disorder (MDD, red). Group difference is significant (Mann–Whitney p = 5.9 × 10^−4^), with a large effect size (Cliff’s δ ≈ 0.94).

**Figure 10:**
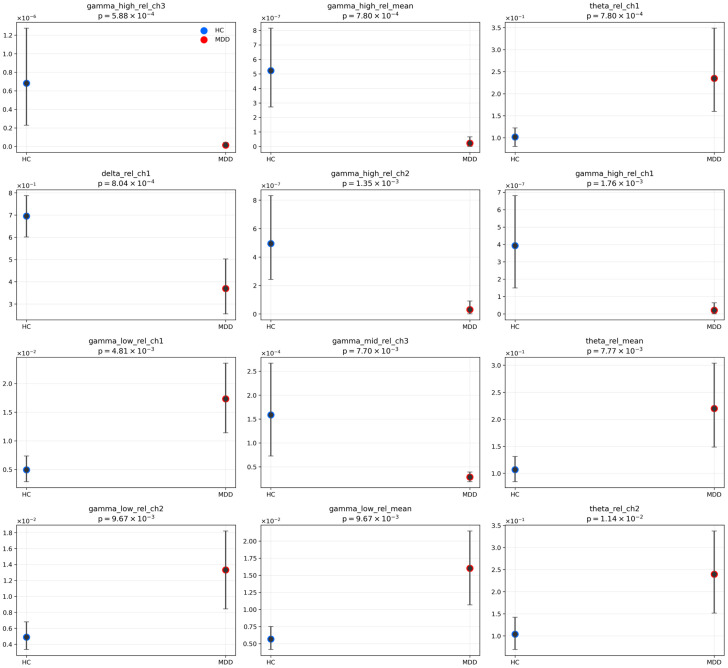
Top subject-level spectral effects across bands and channels in the UNICORN dataset. Mean ± 95% bootstrap confidence intervals for the 12 relative spectral features with the smallest Mann–Whitney p-values, comparing healthy control (HC, blue) and major depressive disorder (MDD, red). MDD shows higher relative theta and low-gamma power and lower relative delta power at Fp1, consistent with a frontal-slowing pattern.

To assess whether the UNICORN recordings are suitable for external validation, we first conducted a descriptive, supporting group analysis (Figure 9 and Figure 10) as a sanity-check of physiological plausibility. For each recording, the three frontal channels were z-scored, the signals were segmented into ≈15 s windows with 50% overlap, and window-level spectral features were aggregated per subject to avoid pseudo-replication. Group differences between participants with major depressive disorder and healthy controls were assessed using two-sided Mann–Whitney tests, and uncertainty was quantified as 95% bootstrap confidence intervals based on 4000 resamples. Among the most pronounced effects was markedly reduced relative high-gamma power in the MDD group (e.g., Fp2 relative high-gamma: p = 5.9 × 10^−4^; Cliff’s δ ≈ 0.94; Figure 9). Additional differences included higher relative theta power in MDD (p ≈ 7.7 × 10^−3^), higher relative low-gamma power (p ≈ 9.7 × 10^−3^), and lower relative delta power at Fp1 (p ≈ 8.0 × 10^−4^) (Figure 10).

Measures based on alpha and beta bands showed smaller, non-leading effects. Overall, the UNICORN sample exhibited a pattern consistent with frontal slowing (elevated theta together with reduced high-gamma), indicating physiological plausibility and supporting the application of the pre-trained hybrid model to these recordings, while recognizing that conclusions are limited by the small sample size.

We evaluated the pre-trained hybrid model on the UNICORN recordings without any re-training. Each recording was passed through the same preprocessing and feature-extraction pipeline as in training—per-recording z-score normalization, segmentation into ≈15 s windows with 50% overlap, and generation of the raw 3-channel windows together with the per-channel spectral–statistical features and the 17-dimensional global descriptor. The model produced a class probability for each window; a per-recording label was then obtained by majority voting across windows, and the corresponding confidence was defined as the mean predicted probability of the winning class:

(3) $${\hat{y}}_{rec}=mode\;\{arg\;\underset{k}{\max}\;p_{ik}\},\;\;\;\;conf=\frac{1}{K}\;\sum_{i\;=\;1}^{K}p_{i},{\hat{y}}_{file},$$

where K is the number of windows in the recording, k∈{HC,MDD} is the class label (healthy control or major depressive disorder), $p_{ik}$ is the model-predicted probability that window i belongs to class k, arg max returns the class with the higher probability for window i, and mode {⋅} selects the class occurring most often across windows (majority vote). Thus, ${\hat{y}}_{\mathrm{rec}}$ is the final per-recording label and *conf* is the vote confidence, defined as the average predicted probability of the voted class across the K windows. Because UNICORN comprises only 20 participants (12 HC, 8 MDD), we report subject-level performance with 95% confidence intervals to reflect uncertainty at this sample size. Using per-recording majority voting across windows, the model achieved 100.0% accuracy (20/20) with a 95% Wilson CI of 83.9–100.0%. Class-wise, specificity (HC recall) was 100.0% (12/12; 95% CI 75.7–100.0%) and sensitivity (MDD recall) was 100.0% (8/8; 95% CI 67.6–100.0%). Given the small cohort, the confidence intervals are wide, and the external evaluation should be interpreted as a preliminary feasibility check.

**Figure 11:**
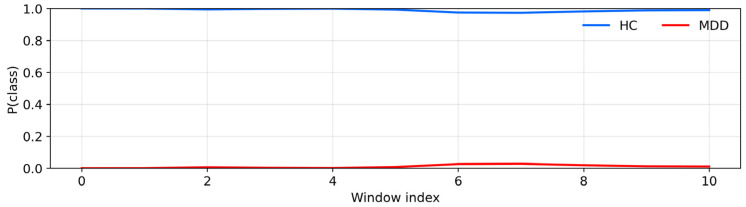
Window-level probabilities for a healthy control (Subject S7).

**Figure 12:**
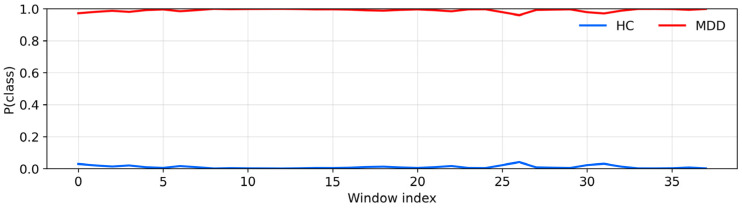
Window-level probabilities for a participant with major depressive disorder (Subject S13).

Across all 20 UNICORN recordings, the hybrid model produced internally consistent subject-level outputs, with vote confidences typically between 0.90 and 0.99. Vote confidence reflects within-recording agreement under the model; subject-level performance is therefore summarized separately above using accuracy and confidence intervals. The window-level curves shown in Figure 11 and Figure 12 correspond directly to the model’s softmax probabilities for each class before majority voting.

To illustrate, Figure 11 shows a healthy-control recording in which the posterior probability of the HC class, p(HC), remains close to 1.0 for all windows, whereas Figure 12 shows an MDD recording with the complementary pattern of near-unity p(MDD) across windows. These examples are representative of the window-level probability traces underlying the majority-vote decisions and associated vote confidences computed for each recording in the batch-prediction summary.

**Figure 13:**
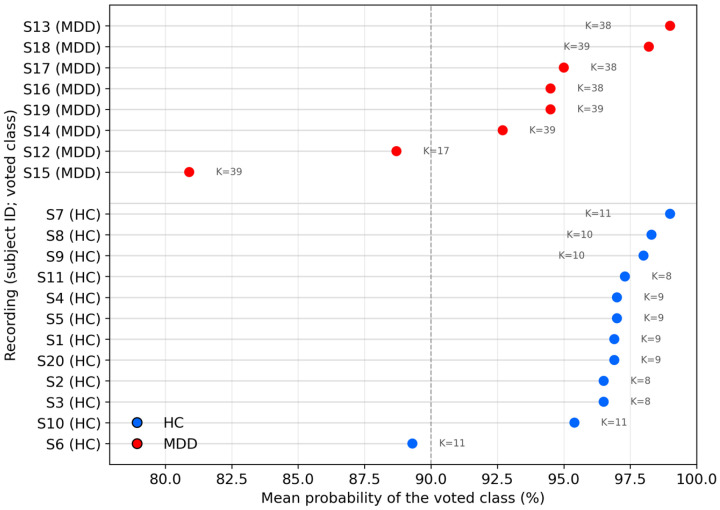
Per-recording decision and confidence on UNICORN. Points show the mean probability of the voted class for each recording; colors encode the voted label (healthy control (HC, blue), major depressive disorder (MDD, red)), and K indicates the number of windows. The dashed line marks 90%. Most recordings fall above 95%, indicating high within-recording agreement under majority voting.

Subject-level predictions were consistently high-confidence across the 20 UNICORN recordings (Figure 13).

Most recordings cluster between 95% and 99% mean predicted probability for their voted class, with only two recordings falling in the 80–90% range (one HC around 89% and one MDD around 81%). The remaining recordings show very strong agreement across windows. Notably, vote confidences remain high even when K is modest, suggesting that subject-level decisions arise from robust aggregation of multiple windows rather than reliance on a single highly confident window.

## 4. Discussion

We introduced a compact hybrid deep architecture that fuses a Conv1D representation of raw ≈15 s, three-channel frontal EEG windows with a lightweight set of per-recording spectral–statistical descriptors. Trained with strict subject-independent splits and modest, label-preserving augmentations, the model reached 93.43% window-level accuracy on the MDD dataset and yielded consistent subject-level decisions via majority voting. When evaluated without any re-training on an independent three-channel acquisition recorded with a different device (UNICORN), the same pre-trained model produced coherent, high-confidence subject-level predictions, indicating that the full preprocessing and modeling pipeline can be transferred across hardware in this small sample. Taken together, these findings support the central hypothesis that low-burden, three-channel resting-state EEG can support MDD versus HC discrimination when paired with a judiciously regularized hybrid model, while motivating further work on larger and more diverse cohorts.

Three-channel depression detection is an active line of research motivated by portability and cost considerations; prior studies have typically relied on hand-engineered features and classical learners, sometimes augmented with advanced transforms or transfer-learning schemes. Our findings are broadly consistent with this literature but add two main contributions: (i) a hybrid raw-plus-features approach that, on the MDD dataset, performs at least as well as purely tabular or purely raw Conv1D pipelines under identical subject-independent splits; and (ii) an external, device-level evaluation using a modern portable amplifier, which, despite the small sample size, illustrates how a model trained on a public dataset can be applied to independent three-channel recordings. The strong performance of simple, physiologically grounded features (relative band powers, ratios, differential entropy) alongside the modest gains from fusing them with temporal Conv1D embeddings highlights that spectral summaries remain informative, while complementary representations can offer incremental improvements in this setting.

To situate our results, Table 5 summarizes a representative (non-exhaustive) set of EEG-based MDD/HC studies spanning classical, deep, and hybrid pipelines, and reports, for each, the classifier type, number of EEG channels, and headline accuracy as given in the original articles. Reported accuracies range from around 72% for three-channel, feature-based k-NN on resting EEG to above 95% for some dense-montage or transfer-learning configurations. However, many of the highest figures are obtained under k-fold or intra-subject validation, or are expressed in metrics such as macro-F1, and are therefore not directly comparable to strict accuracy under subject-independent splits. Relative to other three-channel entries, our hybrid model compares favorably under subject-independent evaluation and is among the few approaches to include device-level external validation, an aspect often missing in prior reports. In the discussion that follows, we therefore interpret performance with attention to differences in evaluation protocol and practical deployment constraints.

Among the three-channel studies that report subject-independent evaluation, our hybrid model is at the upper end of the accuracy range in Table 5 (93.43%). In contrast, the 97.42% result listed for [55] was obtained with 5-fold cross-validation that does not enforce subject separation (i.e., includes intra-subject testing), and is therefore not directly comparable to accuracy under subject-independent splits. Accordingly, our model should be viewed as competitive within the subset of three-channel methods evaluated under strict subject-independent protocols, rather than as definitively outperforming all approaches in the table.

Across our own experiments on the MDD dataset, classical feature-based models and Conv1D architectures also achieved high window-level accuracies (≈89–93%; Table 1), indicating that, for this cohort and preprocessing pipeline, the MDD versus HC distinction is strongly expressed in the data. The hybrid model’s performance gain over these baselines should therefore be viewed as incremental rather than evidence of a qualitatively different decision boundary, especially given the small number of held-out test subjects, for which differences of one to two percentage points are unlikely to be robust across cohorts. Together with the small size of the independent UNICORN cohort and the resulting uncertainty in subject-level estimates, this suggests that our results characterize what is achievable on this specific public dataset under strict subject-independent splits, rather than defining an upper bound for three-channel EEG screening more broadly.

Several design choices likely contributed to the observed generalization. First, we used a strict subject-independent evaluation to avoid leakage from overlapping windows of the same participant. Second, we applied lightweight, label-faithful augmentations (small additive noise, slow drift, amplitude scaling, and limited MixUp) that promote invariance while preserving the underlying spectral structure. Third, we aggregated predictions at the subject level (majority vote with mean probability) to stabilize decisions and provide a practical operating point for screening. In addition, we favored a minimal preprocessing strategy—per-recording z-scoring without ICA/ASR or aggressive offline filtering—to reduce parameter sensitivity and dataset-specific biases, which is particularly important with only three frontal channels, where blind source separation is underdetermined.

Evaluating the pre-trained hybrid model on the UNICORN dataset (Fp1, Fz, Fp2 at 250 Hz) without any re-training served as a preliminary external feasibility check of applying the full pipeline to an independent acquisition without adaptation. Group-level spectral patterns in UNICORN (elevated theta and reduced high-gamma in MDD) were consistent with a frontal-slowing profile and with the frontal spectral patterns observed in the training dataset, suggesting that similar group-level effects are present in this cohort. At the same time, subject-level majority voting on UNICORN yielded mostly high-confidence decisions, with vote confidences typically exceeding 90%. The model footprint (≈40 MB) and the straightforward path to post-training int8 TFLite quantization further suggest that on-device inference in ambulatory or outpatient settings is technically feasible in principle, although this remains to be demonstrated in prospective validation studies on larger cohorts and within appropriate regulatory and clinical frameworks.

In practice, a short eyes-closed recording from three frontal electrodes could be processed by a hybrid model to produce a binary screening suggestion (HC versus MDD) accompanied by a quantitative confidence estimate, supporting triage or longitudinal monitoring in settings where full 19- or 64-channel systems are impractical. Operating thresholds could be tuned, for example, using criteria such as Youden’s index, to favor sensitivity or specificity according to clinical goals, and subject-level aggregation helps reduce the influence of transient artifacts on individual windows. Such tools are best viewed as complements to, rather than replacements for, clinical assessment, and any deployment should incorporate explicit procedures for data-quality checks, confidence reporting, and human-in-the-loop review.

This study has several limitations. Sample sizes remain modest, particularly for external testing (20 subjects), and may not capture the full heterogeneity of MDD phenotypes, comorbidities, or medication effects. The three-channel frontal montage sacrifices spatial coverage and prevents robust artifact separation; although per-recording standardization and subject-level aggregation help, eye and muscle activity can still influence frontal signals. We focused exclusively on resting-state, eyes-closed recordings, so generalization to eyes-open or task-based paradigms remains to be established. In addition, subject-level majority voting aggregates many correlated windows per participant, which stabilizes predictions but can yield subject-level outcomes that differ from window-level metrics. Given the small number of independent test participants, paired statistical comparisons between models are underpowered, and window-level tests are confounded by within-subject correlation under overlap; robust statistical comparison requires larger independent cohorts. Augmentation operators are convenient proxies for acquisition variability but can also introduce non-physiological artifacts [69]; MixUp, in particular, is best understood as an interpolation-based regularizer, and quantifying the sensitivity of results to each augmentation component remains an important topic for larger independent cohorts [70,71].

We primarily reported accuracy and confusion-derived metrics; future work should incorporate calibration curves, decision-curve analysis, and health-economic endpoints to better quantify clinical utility. Finally, although the model could be applied unchanged to a small independent sample, this does not establish external validity across devices or sites; multi-center, prospective evaluations with pre-registered protocols will be needed to quantify robustness across sites, devices, and demographic strata. We also did not benchmark end-to-end deep models trained on higher-resolution spectral or time–frequency representations (e.g., log-PSD curves, STFT/wavelet spectrograms), which typically increase input dimensionality and capacity requirements; benchmarking such approaches under the same strict subject-independent, group-aware protocol is an important direction for future work.

We are considering several directions for future work, including deeper analyses of feature contributions and generalization: (i) prospective, multi-center validation with standardized acquisition procedures and pre-registered analysis plans; (ii) quantifying and, where necessary, improving model calibration (e.g., via temperature scaling) for threshold-based decisions; (iii) investigating on-device inference with int8 quantization and energy profiling; (iv) extending interpretability using saliency or attribution methods over time–frequency representations to verify that decisions rely on physiologically plausible cues; (v) exploring domain-adaptation or post hoc harmonization strategies to improve cross-device consistency; (vi) evaluating the potential of three-channel EEG for longitudinal monitoring, including relapse or treatment-response tracking; (vii) evaluating alternative fusion operators (e.g., logit-level late fusion and lightweight gated/attention-based fusion) to quantify the trade-off between accuracy and model complexity in larger cohorts; (viii) quantifying the marginal contribution of engineered feature groups (e.g., spectral ratios and differential entropy) via group-wise ablation and permutation-based importance analyses on larger independent cohorts; and (ix) assessing the marginal contribution of each augmentation component (noise, drift, scaling, and MixUp) via controlled ablation under the same subject-independent protocol.

## 5. Conclusions

We showed that three-channel frontal resting-state EEG, analyzed with a hybrid deep model that fuses raw Conv1D embeddings with compact spectral–statistical features, can discriminate MDD from HC with 93.43% window-level accuracy under strict subject-independent evaluation and can be applied, without re-training, to an independent three-channel acquisition when using subject-level majority voting in a small external sample. While several baseline models achieve similarly high accuracies on the MDD dataset, the proposed hybrid architecture provides a compact, deployment-oriented design that is technically suited for portable screening workflows. Larger, prospective multi-center studies and on-device validation on more diverse cohorts will be needed to confirm robustness, calibration, and clinical utility in real-world settings.

## Figures and Tables

**Table 1:** Window- and subject-level accuracy on the major depressive disorder (MDD) dataset (held-out test subjects; subject-independent splits). Window-level accuracy is computed over all held-out test windows and summarizes performance under a sliding-window representation with overlapping (correlated) windows. Subject-level accuracy is computed by majority voting across windows within each held-out subject and is treated as the primary indicator of out-of-sample performance because the subject/recording is the independent unit. Because the held-out test cohort is relatively small, subject-level accuracy changes in coarse increments and should be interpreted cautiously. Differences between models are small at this sample size and are reported descriptively; formal significance testing requires larger independent test cohorts.

Model	Augmentations (Training Only)	HP Optimization/Configuration	Window-Level acc. (%)	Subject-Level acc. (%)
Hybrid Deep Fusion Model	Gaussian noise, slow linear drift, amplitude scaling, MixUp	Hybrid architecture combining a raw Conv1D branch with a feature-based MLP on the hand-crafted per-recording feature set (spectral + time-domain). ≈12-trial randomized search over Conv1D/MLP widths, weight decay, dropout, and learning rate on development subjects; final model retrained with the best configuration and ReduceLROnPlateau. Uses 15.36 s (3840-sample) windows and subject-independent splits	93.43	100.00
SVM (RBF)	–	Operates on the standardized 17-dimensional hand-crafted feature vector (spectral + time-domain). StandardScaler; small grid search over C and γ; class_weight = “balanced”	93.43	83.33
SVM + KNN	–	Both base learners use the same standardized 17-dimensional hand-crafted feature vector. Soft-voting ensemble between tuned SVM-RBF and distance-weighted KNN (probabilities averaged, slightly higher weight on SVM)	92.72	100.00
LR	–	Logistic regression on the standardized hand-crafted feature vector; StandardScaler; manual tuning of C/penalty; class_weight used to handle imbalance	92.49	100.00
MLP	–	Two-layer MLP (256–128 hidden units, ReLU) on the standardized hand-crafted feature vector; $l_{2}$ regularization and early stopping; StandardScaler	92.25	100.00
Classical soft-vote (EnsembleSoft)	–	Soft-voting ensemble of LR, RF, GB, SVM, MLP, and LightGBM, all trained on the standardized hand-crafted feature vector with their tuned hyperparameters; class probabilities averaged	91.78	100.00
EnsembleStack	–	Stacking ensemble with RF, GB, SVM, MLP, and LightGBM as base learners on the standardized hand-crafted feature vector and a logistic-regression meta-learner; meta-features obtained from internal validation folds	91.55	100.00
RF	–	Random forest on the standardized hand-crafted feature vector; ≈400–500 trees; tuned maximum depth; class_weight = “balanced_subsample”	90.61	100.00
LightGBM	–	Gradient boosting trees (LightGBM) on the standardized hand-crafted feature vector; tuned number of trees and depth; learning rate ≈ 0.03; class-imbalance handled with class_weight/scale_pos_weight	90.61	100.00
GB	–	scikit-learn GradientBoostingClassifier on the standardized hand-crafted feature vector; ≈300 trees; learning rate ≈ 0.05; other hyperparameters near defaults	90.14	100.00
KNN	–	Distance-weighted k-nearest neighbors on the standardized hand-crafted feature vector; k = 7; Euclidean distance	89.20	100.00
Conv1D-SE (raw)	Gaussian noise, circular time shift, amplitude scaling	Raw 3-channel EEG windows (24 s, 6000 samples). Conv1D backbone with three blocks (64, 128, 256 filters; kernel sizes 11, 7, 5; “same” padding; ReLU; L2 weight decay 5 × 10^−5^), each followed by squeeze-and-excitation (SE) block, max-pooling (pool size 4), batch normalization, and dropout (0.25, 0.35, 0.45). Flatten + Dense(256, ReLU, L2). Adam optimizer with initial learning rate 1 × 10^−3^; ReduceLROnPlateau; up to 120 epochs; batch size 64; subject-independent splits. On-the-fly augmentation with Gaussian noise (σ = 0.02, z-scored units), circular time shifts (±10% of window length), and mild channel-wise amplitude scaling (σ = 0.05), each applied with probability 0.5	92.60	83.33
Conv1D (raw)	–	Raw 3-channel EEG windows (10.24 s, 2560 samples). Three Conv1D blocks (64, 128, 256 filters; kernel sizes 11, 7, 5; “same” padding; ReLU; L2 weight decay 5 × 10^−5^), each followed by max-pooling (pool size 4), batch normalization, and dropout (0.25, 0.35, 0.45). Flatten + Dense(256, ReLU, L2) + dropout 0.50. Adam optimizer with fixed learning rate 8 × 10^−4^; up to 100 epochs with early stopping on validation accuracy; subject-independent splits; no data augmentation and no systematic hyperparameter search	90.87	100.00

**Table 2:** Stage-1 randomized hyperparameter search (short runs with early stopping) on the major depressive disorder (MDD) development subjects. Validation accuracy is the best value reached during each short run. The top-3 short-run trials (4, 6, and 10) were selected for longer retraining in Table 3; the final model corresponds to Trial 4 (Figure 3).

Trial	Val. acc. (%, Validation)	emb_raw	head_units	lr_init	map_drop	map_filters	raw_drop	raw_filters	wd
1	75.80	256	128	0.001	0.35	(48, 96)	(0.25, 0.35, 0.45)	(64, 128, 256)	5 × 10^−5^
2	71.97	128	256	0.0008	0.25	(32, 64)	(0.25, 0.35, 0.45)	(64, 128, 256)	5 × 10^−5^
3	66.56	128	384	0.001	0.35	(48, 96)	(0.25, 0.35, 0.45)	(96, 192, 256)	5 × 10^−5^
**4**	**86.62**	**192**	**384**	**0.0005**	**0.35**	**(48, 96)**	**(0.3, 0.4, 0.5)**	**(96, 192, 256)**	**1 × 10^−4^**
5	72.93	128	128	0.0008	0.35	(32, 64)	(0.25, 0.35, 0.45)	(96, 192, 256)	1 × 10^−4^
**6**	**87.26**	**128**	**128**	**0.0008**	**0.30**	**(32, 64)**	**(0.25, 0.35, 0.45)**	**(96, 192, 256)**	**1 × 10^−4^**
7	85.35	128	256	0.001	0.35	(32, 64)	(0.3, 0.4, 0.5)	(96, 192, 256)	1 × 10^−4^
8	74.84	256	384	0.0005	0.25	(48, 96)	(0.3, 0.4, 0.5)	(64, 128, 256)	1 × 10^−4^
9	65.92	256	128	0.0008	0.30	(48, 96)	(0.25, 0.35, 0.45)	(96, 192, 256)	5 × 10^−5^
**10**	**99.36**	**256**	**128**	**0.001**	**0.25**	**(48, 96)**	**(0.25, 0.35, 0.45)**	**(64, 128, 256)**	**1 × 10^−4^**
11	81.85	192	256	0.001	0.35	(48, 96)	(0.3, 0.4, 0.5)	(96, 192, 256)	5 × 10^−5^
12	78.34	192	384	0.0005	0.30	(48, 96)	(0.25, 0.35, 0.45)	(64, 128, 256)	5 × 10^−5^

Note: Rows in bold indicate the top-3 configurations (Trials 4, 6, and 10) selected for Stage-2 refinement (Table 3).

**Table 3:** Stage-2 refinement runs (longer retraining of the top-3 configurations from Table 2). The final configuration (Trial 4) was selected based on refinement-stage validation performance and training stability.

Trial	Val. acc. (%, Validation)	emb_raw	head_units	lr_init	map_drop	map_filters	raw_drop	raw_filters	wd
**4**	**98.73**	**192**	**384**	**0.0005**	**0.35**	**(48, 96)**	**(0.3, 0.4, 0.5)**	**(96, 192, 256)**	**1 × 10^−4^**
6	97.45	128	128	0.0008	0.30	(32, 64)	(0.25, 0.35, 0.45)	(96, 192, 256)	1 × 10^−4^
10	74.20	256	128	0.001	0.25	(48, 96)	(0.25, 0.35, 0.45)	(64, 128, 256)	1 × 10^−4^

Note: The row in bold indicates the final selected configuration (Trial 4).

**Table 4:** Window-level performance of the hybrid model on the major depressive disorder (MDD) test set.

	Precision	Recall	F1-Score	Support
HC	0.90	0.96	0.93	194
MDD	0.96	0.91	0.94	232
accuracy			0.93	426
macro avg.	0.93	0.94	0.93	426
weighted avg.	0.94	0.93	0.93	426

**Table 5:** Comparative summary of selected EEG-based major depressive disorder (MDD)/healthy control (HC) classification studies. For each work, the classifier type, number of EEG channels, and reported accuracy under the authors’ evaluation protocol are shown.

Reference	Classifiers	No. of EEG Channels	Accuracy (%)
Our method	Hybrid Conv1D	3	93.43
[55] *	LightGBM	3	97.42
[56]	KNN	3	72.25
[57]	MRCNN-RSE	6	98.47
[58]	CK-SVM	7	83.64
[59]	KEFB-CSP + SVM	8	91.67
[60]	EEGNet tuned with Optuna	8	93.27
[19]	Wavelet Transform + ML	19	87.5
[61]	CNN	30	~94–95
[62]	sLORETA + GCN	64	85.33
[63]	LR/ML	128	82.31
[64]	TCN/L-TCN	128	~85–87
[65]	CNN + GRU	128	89.63
[66]	Generator + Discriminator	128	92.30
[67]	Custom deep architecture	128	94.72
[68]	1D-CNN	128	97.00

* Study [55] reports intra-subject validation, which is not directly comparable to subject-independent results.

## Data Availability

The datasets analyzed in this study come from two sources. The MDD public dataset is openly available at the repository cited in reference [72]. The UNICORN dataset (our three-channel acquisition) cannot be made publicly available due to privacy and ethical restrictions. De-identified UNICORN data may be shared upon reasonable request to the corresponding author and are subject to approval by the relevant ethics body and the signing of a data-use agreement.

## Abbreviations

The following abbreviations are used in this manuscript:

MDD	Major depressive disorder
EEG	Electroencephalography
HC	Healthy control(s)
ML	Machine learning
SVM	Support vector machine
KNN	K-nearest neighbor(s)
CNN	Convolutional neural network
1D-CNN	One-dimensional convolutional neural networks
RNN	Recurrent neural networks
ResNet	Residual network
CV	Cross-validation
ICA	Independent component analysis
ASR	Artifact subspace reconstruction
Conv1D	One-dimensional convolutional
EC	Eyes-closed
EO	Eyes-open
FFPI	Five-Factor Personality Inventory
DASS-21	Depression, Anxiety and Stress Scale
GDPR	General Data Protection Regulation
µVpp	Amplitude in microvolts peak-to-peak
SD	Standard deviation
$f_{s}$	Sampling rate
NaN	Not a Number
Inf	Infinity
PSD	Power spectral density
DE	Differential entropy
LR	Logistic regression
RF	Random forest
LightGBM	Light gradient boosting machine
MLP	Multilayer perceptron
GB	Gradient boosting
SE	Squeeze-and-excitation
SVM-RBF	Support vector machine radial basis function
acc.	Accuracy
MCC	Matthews Correlation Coefficient
wd	$\mathrm{Weight}\;\mathrm{decay}\;(l_{2}$ regularization coefficient)
TN	True Negative
TP	True Positive
FN	False Negative
FP	False Positive
macro avg.	Unweighted mean across classes
weighted avg.	Support-weighted mean across classes
p	Probability value
F	females
M	Males
MRCNN-RSE	Multi-resolution CNN with residual squeeze-and-excitation
CK-SVM	Composite kernel support vector machine
KEFB-CSP	Kullback–Leibler-enhanced filter bank variant of common spatial patterns
sLORETA	Standardized low-resolution electromagnetic tomography
GCN	Graph convolutional network
TCN	Temporal convolutional network
L-TCN	Lightweight temporal convolutional network
GRU	Gated recurrent unit
